# Influence of oven-drying pre-treatment on the metabolic profile of extracts from brewer’s spent grains

**DOI:** 10.3389/fnut.2026.1833417

**Published:** 2026-05-29

**Authors:** Martina Minestrini, Simone Zorzan, Marcus Iken, Yvan Larondelle, Jenny Renaut, Jean-François Hausman, Kjell Sergeant

**Affiliations:** 1 GreenTech Innovation Centre, Environmental and Industrial Biotechnologies, Luxembourg Institute of Science and Technology, Rue Bommel, Hautcharage, Luxembourg; 2 Louvain Institute of Biomolecular Science and Technology (LIBST), UCLouvain, Louvain-la-Neuve, Belgium; 3 PM International AG, Schengen, Luxembourg; 4 Wallonia Institute of Food Science and Technology, Charleroi, Belgium

**Keywords:** brewer’s spent grain, residues, up-cycling, metabolomics, anti-oxidant capacity, pretreatment, circular economy

## Abstract

Extracts from untreated (fresh) and treated (oven-dried and ground) BSG, the most abundant residue from the brewing industry, were investigated through UHPLC–MS/MS metabolomic analysis to evaluate the effect of biomass pretreatment and extraction conditions on their chemical composition. Three extraction conditions were applied to each starting material, and the identified compounds were grouped into eight classes based on their chemical structure. The accumulated MS signal intensity of each class of compounds was then compared across the starting materials and the extraction conditions. The results showed that for some compound groups (e.g., sugars, amino acids, and phenolic compounds) drying was the main discriminating factor, while for others (lipids and hordatines) the determining factor was the extraction solvent. In addition, antioxidant capacity assays revealed that extracts obtained from untreated BSG with 40% hydroethanolic solvent displayed the highest activity. Overall, our data indicate that the energy- and time-consuming drying/stabilization pre-treatment step may be avoided. Given the predictable availability of BSG in both quantity and timing this is possible, although confirmation studies at pilot and industrial scale are needed.

## 1 Introduction

Currently, there is a global focus on linking economic development with environmental protection. Therefore, sustainability and re- or upcycling are now considered essential factors in industrial development, and these three concepts must be considered as one entity. This emphasis is further necessitated by the growing population, which reached 8 billion in 2022, and concerns on whether, despite a 53% rise between 2000 and 2019, the global primary production can keep up with the growing demand.^1^

Coinciding with the context described above, the amount of generated organic by-products also increased, leading to a higher quantity of bio-waste to be transformed or disposed (1). The realization that this bio-waste can become resources rather than waste streams, is promoting scientific and societal interest to develop technologies for the circular use of biomass. The general aim is to use agro-industrial residues as renewable sources for producing energy, materials, foodstuffs or to exploit them to extract useful compounds for pharmaceutical, medical and biotechnological purposes (2).

With 1.82 billion hectolitres produced in 2020, beer is the most consumed alcoholic beverage, and brewing represents a highly productive and growing industrial activity. In 2020, European beer production reached 501 million hL (3) resulting in 20 kg of brewer’s spent grain (BSG) for each 100 L of beer. With a total of about 41.2 million tonnes produced annually, BSG is thus the most abundant residue from the brewing process. The low economical value of about 35 euros per ton of BSG (4), its limited valorisation and costs for transport and storage make that the two most common ways to recycle BSG are animal nutrition (70%) and energy production through anaerobic digestion (10%). However, about 20% of the globally produced BSG is disposed of by landfilling (5). On one hand, the current amount of BSG disposed of and its expected increase, due to the rising of beer production, represent one of the biggest concerns of the brewery industry (1, 6). On the other hand, BSG is a residue from an industrial process whose generation is non-seasonal, and its availability is constant and predictable both in amount and timing. These latter features open opportunities to find alternative uses to those mentioned above.

BSG is composed of the non-soluble parts of the grain; husks, pericarp and seed layer-covering. It globally contains protein (19–30%), hemicelluloses (20–25%), cellulose (12–25%), lignin (12–28%), lipid (10%), phenolic compounds, vitamins and minerals (7, 8). Due to the richness of its chemical composition, other approaches for its upcycling, with higher valorisation potential, are being investigated among the scientific community, such as a biomass for biorefinery, a source of food ingredient or substrate for producing enzymes, bioactive molecules and prebiotics (1, 2). However, it is important to note that according to the variety of the grain used as well as the type of brewery process parameters applied, its chemical composition can vary (2, 9, 10).

Current research on BSG valorisation focuses on the recovery of multiple bioactive fractions, including protein hydrolysates with reported anti-inflammatory, antimicrobial and antidiabetic properties (11, 12); polysaccharides such as arabinoxylans and beta-glucans investigated for their prebiotic potential (13, 14) and lipid fractions associated with health-promoting activities (15, 16). Among these, phenolic compounds represent the most extensively studied fraction, owing to their abundance and well-documented antioxidant and health-promoting activities, and constitute the primary focus of the present study. Among those metabolites, hydroxycinnamic acids (HCA) and flavonoids are the most frequently targeted. In fact, BSG consists mainly of the outer layers of barley grains, which are rich in cell walls, where phenolic compounds accumulate. These compounds may occur in either free or bound forms. Free phenolics are predominantly flavonoids, including catechins and their oligomers, quercetin, and myricetin, whereas the bound fraction is largely composed of HCAs, such as ferulic, p-coumaric, sinapic, and caffeic acid (17–19). In addition, a distinct group of barley-specific metabolites (20), known as hordatines, has been identified in BSG and together with precursors dominate the metabolic profile of barley roots and shoots (21). Hordatines are cyclic condensation products of agmatine and HCA monomers. Although derived from phenolic precursors, they represent a separate class of bioactive compounds and cosmetic products are marketed as hordatine containing. Despite their abundance and potential, hordatines are not yet considered quality markers in BSG extraction processes, unlike phenolic metabolites such as ferulic acid, p-coumaric acid, or catechins. All these compounds are known to have a positive effect on human health, making that BSG is considered as a valuable source and the extraction of these compounds a promising approach for BSG valorization (22–24).

Many studies focused on the optimization of the extraction of phenolic compounds from BSG, with the antioxidant capacity of the obtained extracts used as biochemical parameter to select the optimal extraction approach. However, prior to extraction plant-based materials are most often subjected to pre-treatments (25), regardless of the exact extraction approach followed: from conventional techniques such as Soxhlet, maceration and hydrodistillation (26), to more innovative approaches, like ultrasound assisted, microwave assisted, or super critical fluid extractions (27). Actually, most studies do not use raw BSG as such, but apply drying and grinding as pre-treatments (28–30). Drying is considered essential for BSG management as it prevents microbial growth, limits biomass degradation, and reduces weight and volume for transport and storage (31). Lyophilization is considered the best drying technique to preserve the chemical properties of the used biomass as this treatment occurs at low temperatures (25). However, the energy needed and cost and time-consumption for lyophilization, make this method less suitable for industrial BSG valorisation (32–34). Oven-drying is more cost-efficient but requires exposure to high temperatures and long drying times. It has a high energy consumption and, because of the high temperatures, influences the quality/composition of the extract (25, 31, 33). Nonetheless, the detailed effects of oven-drying for industrial BSG valorisation are not known. The pre-washing treatment (also referred to hydrothermal treatment based on liquid hot water) is mostly used on BSG for recovering high-molecular-weight fibers like cellulose, *β*-glucan, and arabinoxylan (35). Furthermore, grinding is frequently used to homogenize the biomass and facilitate the extraction process (36). However, omitting the drying step can be considered a viable alternative, offering a more sustainable approach aligned with circular bioeconomy principles and contributing to a greener, more resource-efficient industrial economy, as it preserves heat-sensitive metabolites while reducing energy consumption and operational costs.

Exposure of biomass to high temperatures during oven-drying can reduce antioxidant capacity and phenolic content via thermal degradation, with lower levels of key phenolics reported in dried BSG extracts (25, 31, 37). In addition, heat-treatment of organic material promotes heat-triggered reactions, like the Maillard cascade, thereby modifying the composition of the extracts (38). The first stable products formed during the early stage Maillard reaction are known as Amadori compounds, products that are formed by a condensation between a reducing sugar and a free amino group (39–41). The formation of Amadori products results in the decreased abundance of sugars and amino acids in extracts from dried products compared to the fresh extracts (42–45). Similarly, drying has been shown to have an impact on the polyamine and lipid content of extracts from plant matrixes (46, 47). In the most detailed study on the effect of drying of BSG so far (48), drying was shown to cause changes in the protein and lipid content of BSG extracts although only the impact on the lipids was significant. However, contrary to data on other matrices, freeze- nor oven-drying had a significant impact on the total phenolic content of extracts from BSG (48).

In the current work, the scientific question whether BSG drying can be omitted without negatively impacting currently used quality parameters for BSG extracts was addressed. To this end a detailed analysis of the metabolite profile of BSG extracts was performed to evaluate the impact of the most commonly used BSG drying method (oven-drying at 50 °C for 48 h). Considering that some compounds may be heat-labile and undergo chemical alterations upon heat treatment, we hypothesized that oven-drying significantly alters the composition of BSG extracts, potentially affecting their bioactive properties and the valorization potential. To obtain a comprehensive view of the drying impact and avoid focusing on individual compounds, the identified metabolites were grouped into eight classes: sugars, amino acids, Amadori products, peptides, hordatines, polyamines, phenolic compounds, and lipids. The impact of drying was then assessed based on the total MS signal of each class, allowing a systematic evaluation of compound distribution and overall trends within each metabolite class as was previously done for other extracts (49, 50). Since the increased formation of Amadori products due to heat treatment was previously linked to decreasing levels of reducing sugars and free amino acids (44), it is hypothesized that the effects of drying on these three compound groups will be correlated. Furthermore, previous studies indicate increased formation of short peptides and their cyclization products in extracts from heat treated biomass. Since a similar trend may be expected in this study, these compounds were added as separate groups as were polyamines. The main activity targeted in BSG extracts is antioxidant capacity, an activity mainly related to BSG phenolic compounds. Data on the effect of drying on the abundance of phenolic compounds in extracts from different biomasses is ambiguous, with both decreases and increases mentioned in literature and a non-significant decrease was previously reported in BSG extracts (48). Although hordatines are phenolic compounds, their barley-specific presence, unique chemical structure and promising bioactivities, justify treating them as a distinct group (20, 22–24). Roasting of barley grains was previously shown to result in a decreased hordatine content (51), however no data is available for the impact of a prolonged, relatively mild heat treatment. The final group contains the lipids. For lipids the abundance is hypothesized to be mainly influenced by the used extraction solvent, although a decreased abundance after drying was previously observed (48).

In addition to the detailed untargeted metabolomic profiling of the extracts, the antioxidant capacity and total phenolic content (TPC), respectively determined with the FRAP assay and Folin–Ciocalteu method, were measured as these represent the biochemical parameters most widely employed for the quality assessment of BSG extracts. The comparative analysis of extracts from fresh and oven-treated BSG was performed by calculating the relative accumulated MS-signal intensity for each defined compound class, following an approach previously performed for other extracts (49, 50). Prolonged exposure to oxygen at elevated temperatures during oven-drying is anticipated to promote oxidative degradation of antioxidant compounds, whereas the net effect on total phenolic content remains less predictable, as it is dependent on the thermostability of individual phenolic constituents.

## 2 Materials and methods

### 2.1 Chemicals

The chemicals mentioned below were purchased. Ethanol (VWR, 20821.321); formic acid (Sigma-Aldrich, 56302); acetonitrile (Sigma Aldrich, 271004-100ML); Folin–Ciocalteu reagent (Sigma Aldrich, F9252-100ML); Na_2_CO_3_ (Sigma Aldrich, 223530-500G); gallic acid (Sigma Aldrich; 27645-250G-R); Hydrochloric acid (HCl) 0.1 M (ChemLab, 31955.373); Iron(III) chloride hexahydrated (FeCl_3_ * 6 H_2_O) (Merck VWR, 1.03943.250); Iron (II) sulfate heptahydrate (FeSO_4_ * 7 H_2_O) (VWR; 24244.232); Sodium acetate trihydrate (C_2_H_3_NaO_2_ * 3 H_2_O) (Sigma Aldrich, 331058-100G); Glacial acetic acid (CH_3_COOH) (J.T. Baker, 9524-33); Fluorescein (Sigma-Aldrich, 46955-100G-F); Trolox (Sigma-Aldrich, 238813-1G); 2,2′-Azobis(2-amidinopropane) dihydrochloride (AAPH) (Sigma-Aldrich, 440914-25G); Potassium phosphate monobasic (KH_2_PO_4_) (Merck, 1.04873.0250); Potassium phosphate dibasic (K_2_HPO_4_) (Vel VWR, PROL26931.263).

### 2.2 Materials

A local brewery, Brasserie Simon Sarl, Wiltz, in Luxembourg, provided a single batch of wet barley-Pilsener BSG and it has been stored in two different conditions: dried and wet. The drying process consisted of placing BSG in an air convection oven at 50 °C for 48 h as previously done (52). After drying, the BSG was milled (Waring Lab Blender) for 2 min at highest speed (18000) on ice and stored at 21 °C under vacuum avoiding light sources. The wet BSG was stored as such under vacuum at −20 °C. For conducting the extractions water and aqueous ethanolic mixtures were used as solvents.

### 2.3 Solvent extraction procedure

Two preliminary experiments were performed for selecting the starting material and the solvent mixture to use. The first preliminary test was conducted on oven-treated, ground (48 h at 50 °C) BSG and untreated, fresh BSG using water and different aqueous ethanolic mixtures (from 0 to 100% of ethanol) as solvent. Extractions were performed at a solid-to-solvent ratio of 1:20 (w/v) in 2 mL tubes, under constant agitation using a bench-top orbital shaker set to 1,400 rpm for 4 h at 21 °C. The procedure was conducted following a previously published protocol (53). Subsequently, based on the data reported on the preliminary test in the Results and Discussion section, 40% of ethanol was selected as the highest percentage of the organic phase for the extraction solvent. Moreover, a second preliminary experiment was performed using untreated fresh BSG and ground fresh BSG using 0, 20 and 40% of aqueous ethanolic solvent. This last test did not highlight remarkable differences between the two types of fresh BSG used. Since this article is part of a wider project aiming at the valorisation of BSG as such, according to the results from the trials mentioned above, the starting material used in the presented study was fresh, untreated, not ground (also referred to as wet) BSG and ground oven-treated (also called dried) BSG.

The experiment described in this study involved two batches of samples: untreated BSG and ground, oven-treated BSG. For each batch, 3 different extraction solvents (MillQ water, ethanol 20% and ethanol 40%) were used.

Extractions were carried out at the ratio 1:20 (solid:solvent) (53) using 3 different solvents: (a) MillQ water (b) 20% of ethanol (v/v%) and (c) 40% ethanol (v/v%) (Table 1) in 2 mL tubes under agitation (Bench-top Orbital shaker) at 1400 rpm for 4 h at room temperature (21 °C).

**Table 1:** Scheme of the experimental conditions and the sample annotation: W indicates extracts from wet, untreated BSG; D extracts from oven-treated and ground BSG.

Sample ID	Experimental conditions
W_MQ	Fresh BSG → extraction with MillQ water (1:20 solid: volume)
W_20	Fresh BSG → extraction with 20% ethanol (1:20 solid: volume)
W_40	Fresh BSG → extraction with 40% ethanol (1:20 solid: volume)
D_MQ	Fresh BSG → oven-drying and grinding → extraction with MillQ water (1:20 solid: volume)
D_20	Fresh BSG → oven-drying and grinding → extraction with 20% ethanol (1:20 solid: volume)
D_40	Fresh BSG → oven-drying and grinding → extraction with 40% ethanol (1:20 solid: volume)

The annotations MQ, 20%EtOH and 40%EtOH, respectively, indicate extracts obtained with MilliQ water, 20% EtOH and 40% EtOH in a 1/20 BSG/solvent ratio.

After extraction, samples were centrifuged for 25 min at 15 °C at 20000 *g* and the upper phase was collected. For UPLC-ESI-MS/MS analyses, 300 μL of supernatants were taken and dried under vacuum (CentriVap; Labconco, Kansas City, MO, United States). The dried residue was resuspended in 300 μL 5% methanolic aqueous solution and filtered with 0.22 μm PTFE syringe filters (Millex-LG; Merck KgaA, Darmstadt, Germany). The filtered supernatants of each sample were stored at −20 °C for biochemical analyses. Four independent extractions were made for each condition.

### 2.4 UPLC-ESI-MS/MS identification

An Acquity UPLC I-class ultra-high pressure liquid chromatography (UPLC) system (Waters, Milford, MA, United States) coupled to an electrospray ionization high-resolution mass spectrometer (TripleTOF 6,600+; SCIEX, Framingham, MA, United States) working in positive and negative modes was employed for injecting 10 μL of each BSG extract. The solvents used were formic acid 0.1% in MillQ water (A) and formic acid 0.1% in acetonitrile (B). The applied gradient was: 1% B at 0 min; 1% B at 4 min; 5% B at 16 min; 40% B at 35 min; 100% B at 45 min; 100% B at 50 min; 1% B at 54 min; and finally, 1% B at 60 min. The column employed was a reverse-phase Acquity UPLC BEH C18 column (2.1 × 100 mm, 1.7 μm particle size, Waters, Milford, MA, United States) and the temperature set at 50 °C, while the flow rate was maintained at 0.5 mL/min.

The ion spray voltage was set at - 4.5 and 4.5 kV, respectively, for the negative and positive mode, an application of 30 psi for curtain gas (nitrogen), of 55 psi for nebulizer gas (air), and for 50 psi for turbine gas (air), with a source temperature of 650 °C. The declustering potential was fixed at −60 in negative and at 60 V in positive mode. For the generation of MS^2^ spectra in high sensitivity mode, the fragmentation of the 10 most intense single-charged precursor ions were employed, to reach a threshold of 100 counts per s, with an accumulation time of 200 ms. The complete cycle lasted 2.225 s and for all precursor ion a sweeping collision energy of −15 V or 15 V in negative and positive mode was set, respectively. Before the subsequent fragmentation, the dynamic exclusion was set at 2 s after three occurrences. For MS, the spectra were detected from 100 and 2000 mass-to-charge ratio (m/z), while for MS^2^ in the profile mode spectra were visualized from 50 to 2000 m/z.

### 2.5 Biochemical assays

#### 2.5.1 Total phenolic content assay (TPC, Folin–Ciocalteu)

The total phenolic content was determined according to a previously described method (54). For this assay, reagents have been employed at the concentration mentioned hereafter: 1 N Folin–Ciocalteu reagent (diluted from the stock solution, provided by Sigma Aldrich, F9252-100ML) and 125 μL 7,5% w/v Na_2_CO_3_ solution. A specific volume of 125 μL was mixed with 50 μL of each sample and standard dilutions in a 96-well plate. The plate was incubated for 30 min in the dark at 21 °C. The absorbance was measured at 765 nm with a spectrophotometric microplate reader (Spark, Tecan, Männedorf, Switzerland). A calibration curve was built by using the standard solution of gallic acid at these concentrations: 0.004; 0.008; 0.0125; 0.016; 0.025; 0.03; 0.05 mg/mL, (y = 4.9609x + 0.0373 *R*^2^ = 0.9989) and the results of TPC were expressed as gallic acid equivalents (GAE).

#### 2.5.2 Ferric reducing/antioxidant power assay

The ferric reducing power assay exploits the potential of molecules to reduce Fe (III) into Fe (II). This reducing power is proportional to the measurement of the antioxidant capacity. The FRAP assay was performed following the method of Benzie and Strain (55). The reagents were freshly prepared by mixing 300 mM acetate buffer (pH 3.6), 10 mM TPTZ solution in 40 mM HCl, and 20 mM FeCl₃·6H₂O in a ratio of 10:1:1 (v/v/v). A 96-well plate was filled with 190 μL of FRAP reagent and subsequently, 10 μL of extracted samples were added and mixed directly in the plate. After an incubation in the dark at 21 °C for 20 min, the absorbance was measured at 595 nm with a spectrophotometric microplate reader (Spark, Tecan, Männedorf, Switzerland). A standard calibration curve using FeSO_4_ at different concentrations was built: 0; 0.05; 0.1; 0.2; 0.4; 0.6; 0.8; 1 mM (y = 0.6853x + 0.0692 *R*^2^ = 0.9998) and the results were expressed as mM of ferrous equivalent.

#### 2.5.3 Oxygen radical absorbance capacity

The ORAC assay principle is to detect the decay of fluorescence intensity related to the measurement of the potential to scavenge peroxyl radicals. The experiment was performed in a 96-well fluorescence microplate reader (Spark, Tecan, Männedorf, Switzerland) by adapting the method of Gillespie et al. (56). 150 μL of fluorescein 0.08 *μ*M, previously prepared in phosphate buffer 75 mM at pH 7, were mixed with diluted samples (100x), blank and standards. A pre-incubation step of the microplate lasting 10 min at 37 °C was done in the microplate reader, and 25 μL AAPH solution (40 mg/mL) was added. The fluorescence was detected for 60 min during 12 cycles, each 5 min. A standard curve was made using Trolox dilutions (6.25; 12.5; 25; 50 *μ*M) (y = 0.682x, *R*^2^ = 0.9931) and results were expressed as *μ*mol Trolox equivalents.

#### 2.5.4 Glucose assay

Glucose was quantified with the Megazyme Sucrose/D-Fructose/D-Glucose Assay Kit according to the manufacturer’s protocol (K-SUFRG, Megazyme, Wicklow, Ireland). Ten μL of extracts and standard dilutions were prepared and added in a flat-bottomed 96-well plate. Then, 10 μL of Solution 1 (Buffer), and 10 μL of Solution 2 (NADP+/ATP) and 5 μL of Solution 3 (hexokinase and glucose-6-phosphate dehydrogenase) prediluted to 40% were added to each well. A pre-incubation of 30 min at 37 °C was done before the detection. The absorbance was set at 340 nm (A1). Subsequently, 5 μL of Solution 4 (phosphoglucose isomerase) prediluted to 40% were added. The absorbance was measured at 340 nm after 20 min of incubation at 37 °C (A2). A standard curve was built for each measurement at several concentrations from 0 to 2000 mg/L of pure glucose. The linear equations (yi = αxi + βi) of the standard curves were used for quantification.

### 2.6 Data analysis

#### 2.6.1 Compound identification

Compounds were identified analyzing the raw data (positive and negative modes) in Progenesis QI (v2.3, Nonlinear Dynamics, Newcastle upon Tyne, United Kingdom) as previously described (41). Compounds (defined as RT x m/z couples) without MS/MS spectra and suspected and known contaminants were filtered out. Subsequently, MS/MS spectra were used for identification. Initial identifications were obtained using our in-house database, containing the data of all identified compounds at LIST since 5 years. Additional identifications were obtained through comparison of MS/MS spectra with data available in literature or databases: HMDB^2^; LipidMaps^3^, GNPS^4^; MZCloud^5^; PubChem^6^ and Metlin.^7^ For other compounds, theoretical spectra generated with MetFrag^8^ and CFM-ID^9^ were used for comparison. A newly identified compound was added in an in-house tool for dereplication and database-generation, that can be exported as msp-file, compatible with Progenesis. All compounds were identified at level 2 based on the m/z-value of the precursor and spectral matching of the fragmentation spectra (MS/MS) with experimental or theoretical MS/MS spectra (57, 58).

#### 2.6.2 Data treatment

The moisture content of BSG was determined by weighting BSG before and after 48 h at 50 °C in an air convection oven. A preliminary study showed that a constant weight, corresponding to a weight loss of 75%, was obtained after 48 h of drying in an air convection oven. Since all extractions were done on 75 mg of biomass, treated or untreated BSG, a factor four was used in all comparisons of the different starting materials (36). This factor was also used in the comparison of the signal intensity of UPLC-ESI-MS/MS measurements.

Wet and dried BSG extracts were analyzed by applying an untargeted metabolomic approach with UPLC-ESI-MS/MS, wherein the relative abundance of each compound based on its MS signal intensity was evaluated. Then, the resulting data were examined with Progenesis QI. Compounds were identified, and their relative abundances were calculated for all the 6 experimental conditions as previously described (50). All identified compounds were grouped into eight classes (sugars, amino acids, Amadori products, peptides, hordatines, polyamines, phenolic compounds and lipids) based on their chemical and functional properties. Data were normalized to sample weight, log-transformed, and Pareto-scaled (mean-centered and divided by the square root of the standard deviation of each variable) using MetaboAnalyst 5.0.^10^ Missing values were imputed using a minimum-value replacement approach (1/5 of the minimum positive value), and variables were filtered based on interquartile range criteria. Statistical analysis included PCA for exploratory data analysis and hierarchical clustering heatmaps (Euclidean distance; Ward’s and average linkage). Univariate differences were assessed using one-way ANOVA (*p* < 0.05) followed by Fisher’s LSD post-hoc test.

Four replicates were measured in technical triplicates for FRAP, ORAC, TPC and the glucose assay and the data analyzed using one-way ANOVA with Tukey’s post-hoc test (*p* < 0.05).

## 3 Results and discussion

The present study investigates whether oven-drying of BSG prior to extraction affects extract composition and valorisation potential, using a comprehensive metabolomic approach and evaluating both metabolite profiles and antioxidant capacity in extracts from fresh and dried BSG. So far, limited studies have used fresh BSG for extraction; Santos et al. (48) compared lyophilised, oven-dried and fresh BSG but found significant differences only in lipid content, while a more recent biorefinery study used fresh BSG without comparison to dried biomass (59).

To evaluate the impact of oven-drying on the metabolic profile of BSG extracts, an untargeted UHPLC-ESI-MS/MS metabolomic approach was applied to extracts from fresh untreated and oven-dried ground BSG. In line with previous observations on other plant matrices (48, 59), oven-drying appearing to induce compound class–specific effects on extract composition, with the starting material emerging as the main discriminating factor across most compound groups, as suggested by PCA graphs.

### 3.1 Preliminary tests

Extractions with 0 to 100% of ethanol (v/v %, water/ethanol %) on ground oven-treated, and fresh BSG were conducted, and the antioxidant potential and TPC of these extracts measured. Among the extracts from oven-treated BSG, 60%EtOH resulted in the highest values for both parameters. While 40% EtOH extracts from fresh samples gave the highest values. Comparing both batches, extracts from fresh BSG resulted in a higher TPC and antioxidant capacity (Supplementary Figure 1). Lower organic solvent concentrations were associated with enhanced extraction efficiency in fresh samples. The reduced antioxidant potential of oven-dried biomass is likely attributable to thermal degradation and structural transformation of heat-labile phenolics during drying, resulting in decreased phenolic content and antioxidant activity. In particular, reductions in phenolic compounds, such as p-coumaric acid, catechins, and procyanidins, considered as quality markers in BSG extracts, have been reported after drying, consistent with thermally induced degradation and loss of bioactive constituents (25, 31, 37).

Since the overall highest FRAP and TPC values were obtained for the extraction with 40% EtOH on fresh BSG, and these parameters are generally used for initial process optimization in the valorization of BSG, 40% EtOH was selected as highest organic solvent composition in the following trials. In a second preliminary test, extracts with water, 20 and 40% ethanol from fresh untreated BSG were compared to extracts from fresh, ground BSG. Grinding did not show a significant impact on antioxidant capacities and TPC, therefore the starting materials selected for this research were oven-treated, ground BSG and fresh untreated BSG.

### 3.2 Analysis of MS intensity signals among the categories of identified compounds

UHPLC-ESI-MS/MS analyses have been carried out on extracts from untreated and ground, oven-treated BSG. The main compounds were identified and grouped in 8 classes according to their chemical nature and properties. The sum of the MS signals of all compounds in a class was used to compare the different experimental conditions. Although this approach does not provide absolute quantification of every class or of specific compounds, it provides information on the general impact of the sample treatment on the global composition of the BSG extracts, providing a clear comparative framework between fresh and dried biomass.

**Figure 1:**
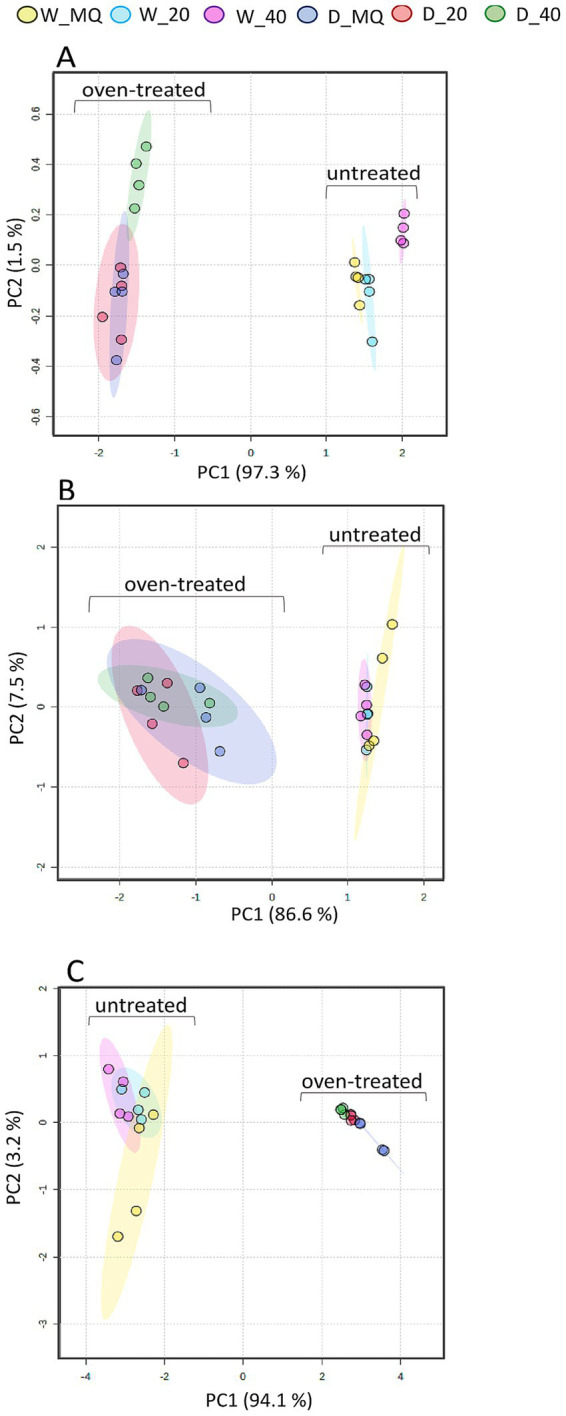
Principal component analysis (PCA) of: **(A)** sugars; **(B)** Amadori products; **(C)** amino acids. W indicates extracts from wet, untreated BSG; D indicates extracts from ground oven-treated BSG. The annotations MQ, 20 and 40, respectively, indicate extracts obtained with MillQ water, 20% EtOH, and 40% EtOH. In all the present graphs, PC1 covers most of the variation among the samples showing a clear segregation between the two conditions: oven-treated and untreated. In the graph **(A)**, samples extracted with 40% ethanol in both oven-treated and untreated conditions resulted in a higher variation compared to the MillQ water and 20% ethanol extracts, while in the graphs **(B,C)**, the three solvent mixtures presented a similar trend.

Overall, oven-drying significantly reduces the abundance of free sugars. The summed MS-signal intensity of the identified sugars was higher in untreated BSG extracts compared to those from oven-treated BSG, which is consistent with studies conducted on malt (60, 61) and agrees with the hypothesis that Maillard reactions contribute to the reduced abundance of free sugars. Concomitantly, the relative intensities rose slightly with increasing EtOH concentration. The highest value was obtained with 40% EtOH extracts from fresh BSG. PCA shows that the main segregating factor between those samples is the starting material (PC1 97.3%), and only little variance (PC2 1.5%) is explained by the solvent mixtures used for extraction (Figure 1A). Additionally, a glucose assay was performed confirming those data (Supplementary Figure 2). The highest free glucose concentration (13.6 mg glucose/ g dw BSG) was obtained for extracts with 40% EtOH from wet BSG samples. Extracts from dried BSG had lower concentrations but also here the highest values were measured in 40% EtOH extracts. Previously, a range from 0.5 to 4.2% w/w of free glucose was measured in 4 extracts from freeze-dried BSG (61), while a value of 1.3 mg glucose/ g was determined in oven-dried BSG (62). Moreover, Kumari et al. found 3.33% glucose in light BSG versus 0.5% in dark BSG (44). These authors related the lower free glucose concentration in extracts from dark BSG with the increased occurrence of heat triggered reactions caused by a more intense kilning process for obtaining dark BSG. It was hypothesized that the decrease in free glucose concentration may be due to the generation of Amadori rearrangement products. These compounds are formed during the early stage of Maillard cascade from a condensation between a free amino group with a reducing sugar of which glucose is the most abundant in BSG (44). As dark BSG, the dried biomass analyzed in this study was subjected to a heat-treatment which may have contributed to the generation of Amadori products and explain the lower free glucose concentrations in extracts from dried BSG.

This is confirmed in our data, oven-drying of BSG leads to an increase in Amadori products. We have recently shown that Amadori products are not only formed from glucose but also from oligo-saccharides (41), which could contribute to the lower relative MS-signal intensity for compounds here classified as sugars in extracts from oven-treated BSG (10, 44, 63, 64). The Amadori compounds identified in this study included fructosyl-glycation products with p-Glu (pyroglutamic acid), Met, Val, Tyr, Ile/Leu, Phe, Pro and Trp. This is in line with the most abundant Amadori products detected in barley malt which are fructosyl-Val, -Ile/Leu, -Phe and -Tyr (65). The PCA graph showed a separation between the two starting materials, again confirming that the pre-treatment had an important impact on the formation of these compounds (PC1 94.1%) (Figure 1B). Regardless of the starting material, the highest values for Amadori products were obtained in extracts with water, although the impact of the used extraction solvent is low. Fructosyl-Phe and fructosyl-Ile/Leu have already been reported to increase more than 13-fold after drying of BSG (66). Amadori products, previously identified in wheat beer and malt, in barley (67) and in BSG extracts (41), are mainly generated during heating of sugar-rich food such as vegetables, flour-based products and fruits (39, 43, 68). In addition to Amadori compounds, a Maillard reaction cascade product of lysine, called pyrraline (69), was identified and detected with a higher signal intensity in oven-dried BSG samples. This molecule is used as an indicator of heating evaluation, as its abundance correlates with the intensity of heat treatment applied in food processing (70). Together, these results show that oven-drying, even at moderate temperatures, results in a higher abundance of Amadori products, thereby lowering the content in free sugars and potentially altering the bioactivities found in the extracts.

Drying generally decreases the abundance of free amino acids, likely due to their participation in Maillard reactions and the formation of Amadori products. Similar to sugars and Amadori products, in the amino acids category, the PCA analysis shows that the highest variance is caused by the drying pre-treatment (PC1 86.6%) (Figure 1C) Using LC–MS, we only identified a limited number of amino acids: Tyr, Phe, Trp, p-Glu, Glu, Arg, Ile/Leu, Asp. As reported in the Supplementary Figure 3A, their relative abundances were higher in fresh BSG extracts than in those from dried BSG (71). These results demonstrate that thermal treatment leads to a reduction of free amino acids, which are subsequently utilized in the formation of Amadori compounds, thereby promoting their accumulation during heat-induced Maillard reactions. This is similar to what is reported in a study on *Curcuma longa* L. wherein all the analyzed amino acids were higher in the fresh material (50). Tyrosine was the amino acid with the highest MS-signal intensity in both treated and untreated BSG, followed by isoleucine/leucine. Instead, glutamic acid, which was observed in high relative concentration in fresh BSG extracts, showed an important decrease in extracts from dried BSG. Just as roasted and black malts contain less free amino acids than green, pale and lager malts, due to the higher temperature of kilning (71), drying BSG reduces the free amino acid content (50). The generation of Amadori products may contribute to the general decrease in the abundance of the amino acids as proposed previously (44).

**Figure 2:**
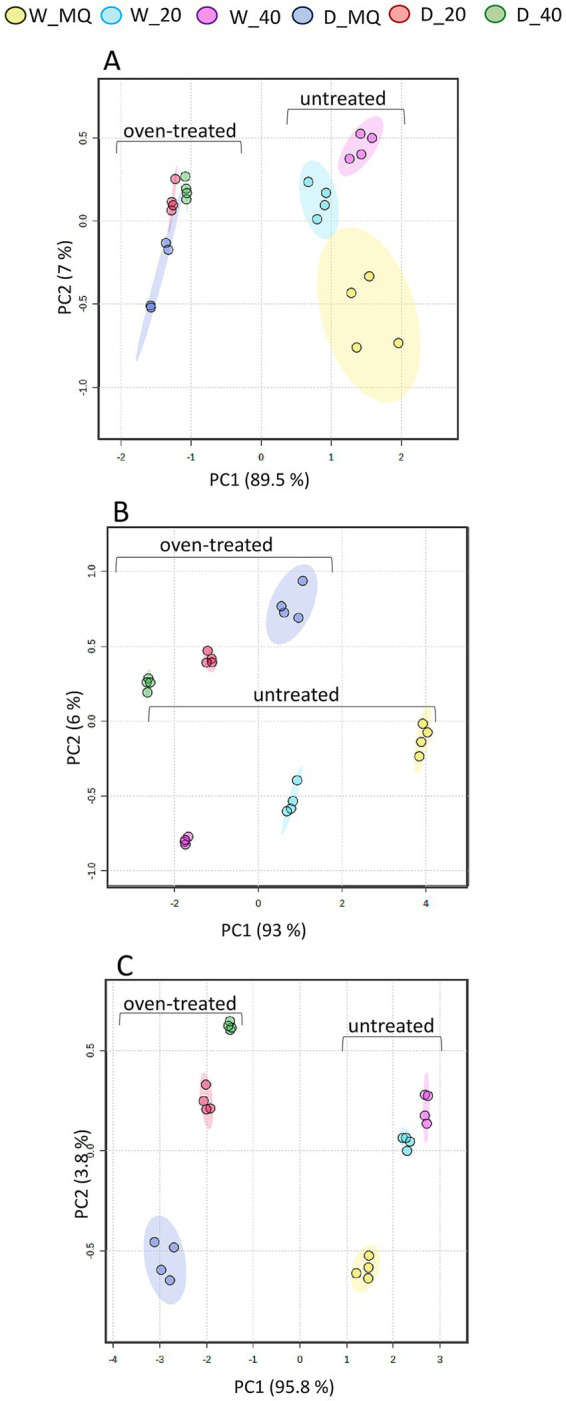
Principal component analysis (PCA) of: **(A)** peptides; **(B)** hordatines; **(C)** polyamines. W indicates extracts from wet, untreated BSG; D indicates extracts from ground oven-treated BSG. The annotations MQ, 20, and 40, respectively, indicate extracts obtained with MillQ water, 20% EtOH, and 40% EtOH. A clear separation is shown in the graph **(A,C)** between oven-treated and untreated samples on the PC1, while on the PC2 the variability is mostly explained by the extraction solvent used. Conversely, the PC1 of graph **(B)** is mainly determined by the extraction solvent used while samples from the different starting biomasses are separated on PC2.

Heat treatment increases the relative abundance of di- and tripeptides and their cyclized derivatives (diketopiperazines), reflecting further protein degradation during drying. Di/tripeptides and derivatives of dipeptides such as diketopiperazines had a higher relative abundance in extracts from oven-dried BSG and extracts from untreated BSG are separated from those of dried BSG on the first PC (Figure 2A). In BSG extracts, the presence of these peptides is expected, since, during mashing the temperature gradually increases from 37 to 78 °C and the enzymatic activity is enhanced resulting in the conversion of proteins into oligopeptides (72). The higher concentration of these compounds in extracts from dried BSG may be related to the further heat treatment during drying that may induce further protein degradation (73–75). During thermal treatments dipeptides can spontaneously form diketopiperazines by cyclisation (76). In this study, thirteen diketopiperazines were identified, twelve containing proline and cyclo (Ile/Leu-Ile/Leu). Due to its ring configuration that stabilizes the compounds during thermal treatment, proline is the main amino acid involved in diketopiperazine formation in beer and processed food (74, 75, 77, 78). The heat-induced enrichment in di- and tripeptides and their cyclised derivatives (diketopiperazines) is of particular interest, as BSG-derived peptides have been reported to display bioactivities including ACE- and DPP-IV-inhibitory and anti-inflammatory effects (79–81). However, these activities have so far mainly been characterized following enzymatic hydrolysis rather than thermal processing, making the bioactive potential of the short peptides released during oven-drying a relevant direction for future research.

Unlike the previously discussed compounds, the total MS-signal sum of hordatines remains largely unaffected by oven-drying. The abundance of these compounds is primarily influenced by the extraction solvent. In the present study, these molecules were detected in higher amounts in extracts with 40%EtOH from both dried and fresh BSG. Although a slight increase in their summed signal intensity is observed after drying, the solvent used for the extractions is the main parameter, represented by PC1 (93%) (Figure 2B). Three different hordatines were identified in this study: hordatine A is the homodimer of p-coumaroylagmatine; hordatine B is a p-coumaroylagmatine and feruloylagmatine heterodimer; hordatine C is formed by the dimerization of 2 molecules of feruloylagmatine. Hordatine A, B and C have also been detected as (di)hydroxylated compounds and glycated hordatine A and B (mono- and disaccharide) have also been identified. Hordatine A has the highest MS-signal in 40%EtOH samples from both materials, covering about 40% of the total MS signal of this group, followed by hordatine B, with 35% of the MS intensity. While abundant in barley and barley products, hordatines have only recently started to gain scientific attention because of their emerging potential benefits for human health (20, 82, 83). Recent studies reported that hordatines exhibit the highest antioxidant capacity among all identified beer constituents (84). Moreover, hordatines contain agmatine, a molecule involved in arginine and polyamine metabolism and currently investigated for its neuroprotective effects in clinical studies, suggesting that hordatines could represent a natural source of this bioactive metabolite (83, 85). Our data shows that, in contrast to other compound groups, thermal treatment has limited impact on hordatines. This stability can be explained by their cyclic dimer structure and conjugated aromatic system, which protects them from thermal degradation.

In contrast to hordatines, the abundance of less complex polyamines, including, dicoumaroylspermidine, *p*-coumaroylspermidine, feruloylspermidine, diferuoylputrescine and the hordatine precursors coumaroyl(hydroxy)agmatine, and feruloyl(hydroxy)agmatine, exhibited reduced MS signal intensities in dried BSG extracts relative to wet samples. Regardless of the type of BSG used, higher signal intensities were obtained from extracts with 40% EtOH compared to the other solvents. The PCA indicates that the initial biomass is the main determinant to separate the samples (Figure 2C). Spermidine, putrescine and agmatine conjugates are ubiquitous in the plant kingdom and have already been identified in barley and barley-based food and beverages, including beer (86, 87). Their content in food varies depending on the processing and storage conditions. Conflicting data on the effects of heat treatments on the abundance of polyamines have been reported. Some studies report that high temperature treatments reduce the polyamine content, while other studies indicate that the polyamine content increases after heat treatment (88, 89). These contradictory results may indicate that for some plant matrices heat treatments increase the extractability of polyamines while for other matrices thermal degradation may be dominant. Some polyamines have been reported to have beneficial activities on human health. An antioxidant activity has been demonstrated for spermidine (89) as well as for polyamines conjugated to hydroxycinnamic acids (90). Specifically, agmatine derivatives of hydroxycinnamic acids have anti-depressant and anxiolytic activity and can be used for treating metabolic diseases (89, 91). Therefore, preserving polyamines in extracts could enhance their bioactive potential, making fresh BSG particularly valuable.

**Figure 3:**
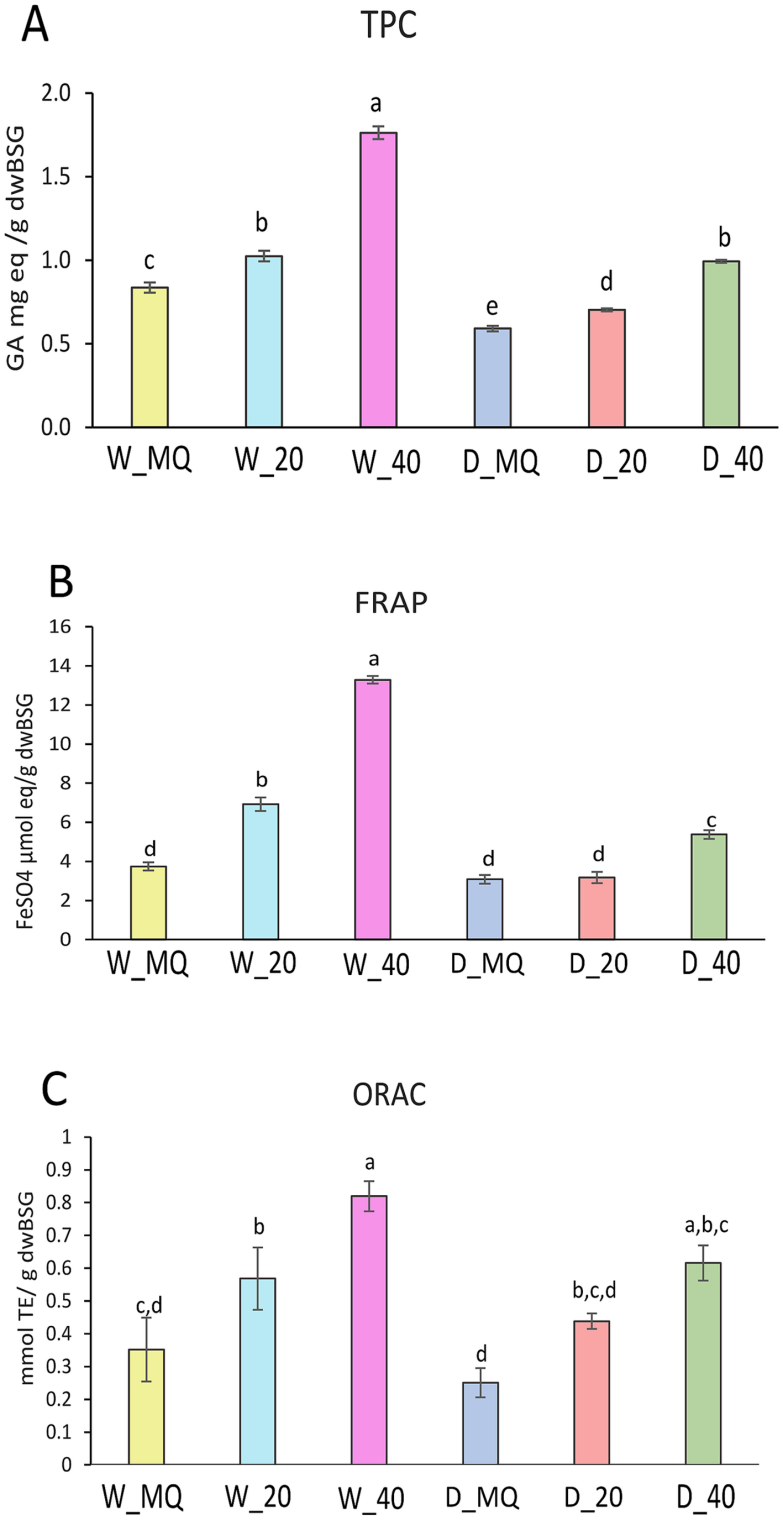
Antioxidant assay on BSG extracts: **(A)**total phenolic content (TPC) (Folin–Ciocalteu); antioxidant capacity as measured with **(B)** FRAP and **(C)** ORAC. W indicates extracts from wet, untreated BSG; D indicates extracts from ground oven-treated BSG. The annotations MQ, 20, and 40, respectively, indicate extracts obtained with MillQ water, 20% EtOH, and 40% EtOH in a 1/20 BSG/solvent ratio.

For the group of compounds referred to as phenolic compounds, a comprehensive list of which is added in a heatmap in Supplementary Figure 3B, extracts obtained with 40%EtOH gave the highest relative MS signal for both untreated and treated starting biomass. For most of these compounds, the MS intensities increased gradually from water to 40%EtOH samples (Supplementary Figure 3B). This agrees with the data from the TPC, FRAP and ORAC assays (Figure 3). These results confirm that solvent polarity plays a crucial role in extracting phenolic compounds, whereas thermal treatment tends to reduce their abundance. The TPC of untreated and oven-treated BSG extracted with 40%EtOH was 1.97 and 1.19 mg GAE eq./g dw BSG, respectively. For the other extraction solvents, lower values were obtained, e.g., 0.85 mg GAE eq./g dw BSG for wet and 0.67 mg GAE eq./g dw BSG for dried samples extracted with 20%EtOH. Although water extractions showed the lowest TPC, these values are still higher than those reported by Connolly, using fresh BSG (92). The lower TPC in the study mentioned above may be justified by the different solid (biomass content)/solvent volume ratio and shorter extraction time applied. The decrease in TPC from 40% EtOH to water was previously observed (53). Our results for the 40% EtOH extracts are in the TPC value range (from 1.00 mg GAE eq./ g dw BSG (23) to 3.3 mg GAE eq./g dw BSG (22)) reported in literature. The antioxidant capacity obtained with the FRAP and ORAC assays are in line with the trend of the TPC; 40% EtOH extracts have a higher antioxidant capacity than 20%EtOH and water extracts with respectively: 14 μmol FeSO_4_ eq/g dw BSG for wet BSG, 5.37 μmol FeSO_4_ eq/g dw BSG for dried BSG (FRAP) and 0.81 mmol TE/ g dw for wet BSG and 0.61 mmol TE/ g dw BSG dried (ORAC) (Figure 3). These are higher than FRAP and ORAC values previously reported (53, 93).

The TPC and the antioxidant capacity measured with FRAP and ORAC are higher in extracts from untreated BSG compared to those obtained from oven-dried BSG. The differences between these extracts are significant, contrary to a previous study which found small, non-significant differences in TPC between extracts from wet, oven- and freeze-dried samples (48). While little data are available for extracts from fresh BSG, comparisons between the use of dried and fresh biomass have been made for other plant-based resources. As previously reported (94), drying decreases the antioxidant capacity and TPC of spearmint extracts. A similar observation was made for extracts from black grape (95). Furthermore, analysis of fruits such as apricots, cranberries, guava, mango and watermelon (96–98), quinoa and cereals such as red rice (99, 100) resulted in a higher TPC in extracts from fresh compared to heat-treated biomass. The mechanisms behind these decreases in antioxidant capacity and TPC have been studied in multiple biomasses and are attributed to direct oxidation, thermal degradation and the formation of complexes with other compounds such as proteins (101). The relative contribution of these mechanisms depends on factors inherent to the used biomass but also on process parameters. While drying at higher temperatures favors thermal degradation, lower temperatures result in a longer exposure to oxygen, thereby favoring the loss of phenolic compounds and antioxidant capacity due to direct oxidation (102). The methods used for drying biomass have a different impact on the phenolic content of extracts (103). Freeze dried chokeberries showed the highest concentration of selected phenolic compounds, the low temperatures result in a better preservation of the bioactive compounds and thus in a higher antioxidant activity (104). A study on different ginger species confirms that all thermal drying methods result in a decreased antioxidant capacity coinciding with decreased values in the Folin–Ciocalteu assay (105). Overall, this indicates that mild thermal treatment, such as oven-drying at 50 °C, lowers phenolic content and antioxidant activity, though the impact varies according to specific compound and matrix.

The examples in the previous paragraph indicate that thermal treatment, including drying, often results in a in a lower antioxidant capacity and total phenolic content, nonetheless different studies found a higher antioxidant capacity after thermal treatment of biomass. Although drying of plums at 85 °C for 18 h resulted in the degradation of anthocyanins and ascorbic acid and temperature-dependent decreases in chlorogenic and neochlorogenic acid, this coincided with an increased antioxidant capacity in dried fruits (106). This increased antioxidant capacity is attributed to the heat-induced formation of hydroxymethylfurfural (HMF), a Maillard reaction product used as indicator of heat exposure in honey and processed cereal products (107). Piga *et al.* show a correlation between the increased absorbance at 280 nm, the absorption maximum of HMF, and the increased antioxidant capacity in dried plums (106). Our dataset was screened for HMF and other known downstream Maillard reaction products and only pyrraline was identified at a low MS-signal intensity. Furthermore, data on malt confirms that the formation of HMF and other downstream Maillard reaction products requires exposure to higher temperatures than those used here during drying. Hellwig and Henle found that the quantities of free glycated lysine are comparable between light and dark malt. However, with for instance an average HMG concentration of 19 μmol/kg for light malt, 446 μmol/kg for dark malt and 9,000 μmol/kg for roasted malt, downstream Maillard reaction products such as HMG are nearly absent in light malt while abundant when light malt is darkened (67). Dark malt is produced from light malt through a heat exposure at > 200 °C for 4 hours. The temperature used for drying in this study is much lower (50 °C for 48 h) and comparable to those used during the first stages of kilning therefore it can be assumed that during drying little downstream Maillard reaction products are formed. Under our drying conditions (50 °C), the formation of Amadori products (Figure 1) does not result in the formation of compounds that compensate for the antioxidant capacity lost due to direct oxidation or thermal degradation of antioxidants, as was observed in dried plums.

**Figure 4:**
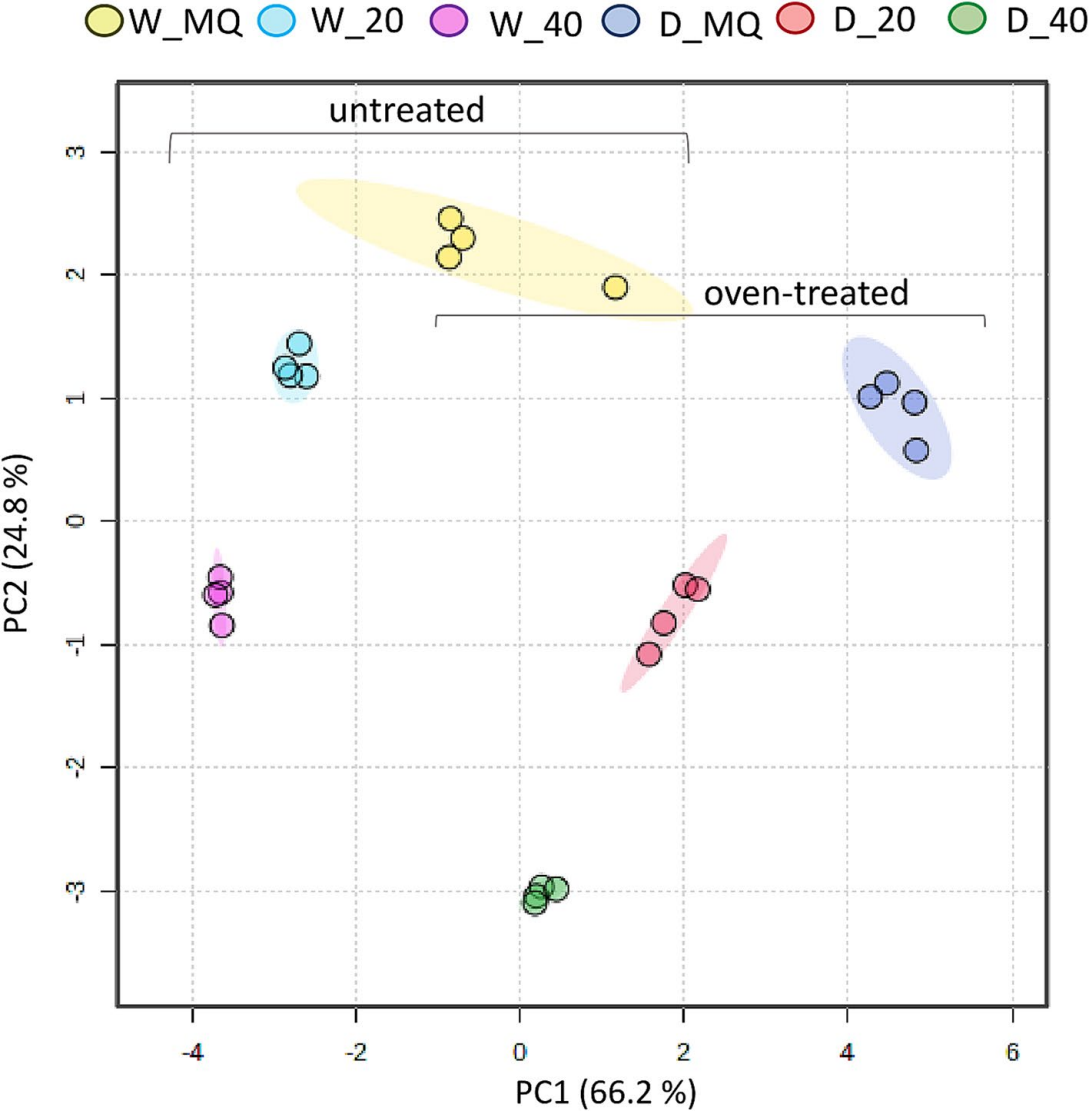
Principal component analysis (PCA) of phenolic compounds. W indicates extracts from wet, untreated BSG; D indicates extracts from ground oven-treated BSG. The annotations MQ, 20 and 40, respectively, indicate extracts obtained with MillQ water, 20% EtOH and 40% EtOH. The variability shown on the PC1 is reconducted to the starting material conditions, although the separation is not sharply defined. The PC2 is clearly determined by the different solvent mixtures wherein both conditions (oven-treated and untreated) present the same trend according to the solvents used.

PCA analysis with this group of compounds indicates that the treatment of the BSG, projected on PC1 representing 66.2% of the variability, is the main determinant of the variation. Nonetheless and contrary to the previously discussed groups, the solvent mixtures used for the extraction also explain 24.8% of the variation on PC2. The impact of the used solvent is similar for untreated and oven-treated samples (Figure 4). This suggests that the abundance of phenolic compounds in the extracts is influenced by extraction solvent and thermal treatment, unlike other compound groups where one factor predominates.

**Figure 5:**
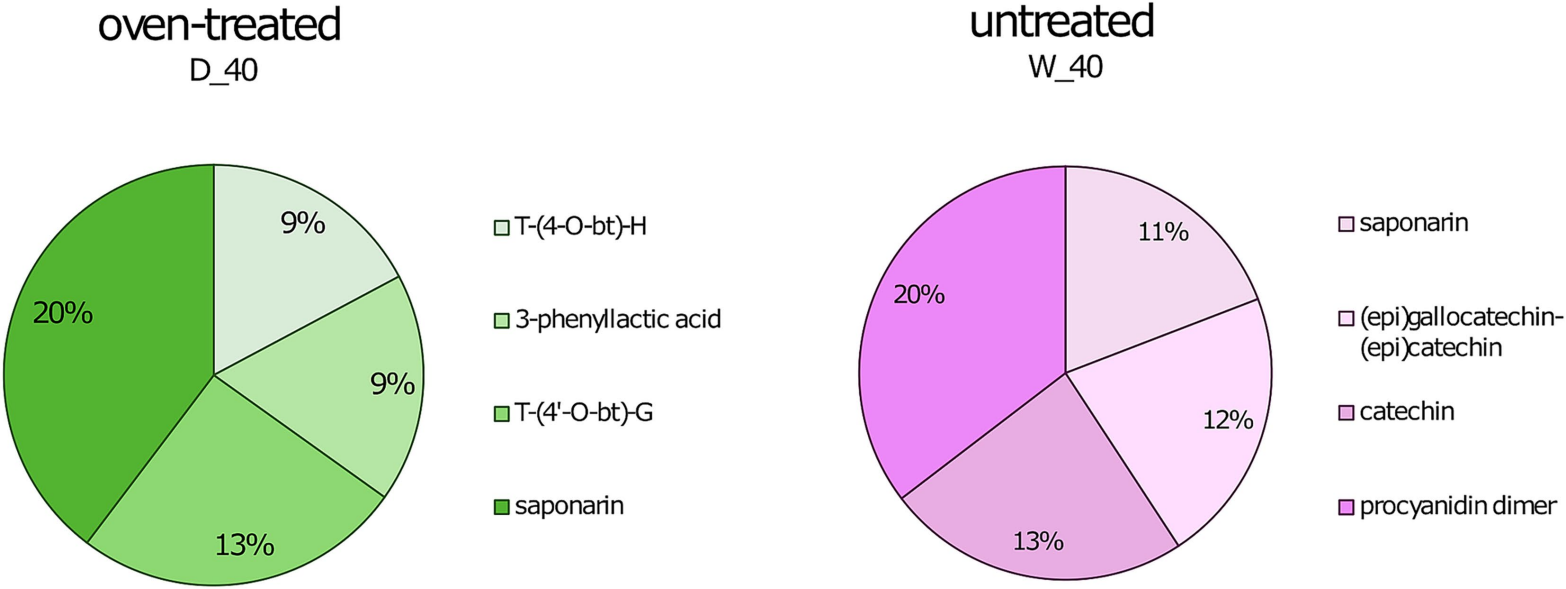
Graph of the most abundant phenolic compounds in oven-treated and untreated BSG. D indicates extracts from ground oven-treated BSG; W indicates extracts from wet, untreated BSG. The annotation 40 indicates extracts obtained with 40% EtOH. The compounds with the highest signal intensity classified in the phenolic group are shown and represented as a percentage of the sum of the signal for the entire group of phenolic compounds. In extracts from oven-treated BSG saponarin and tricin derivatives form the majority, while in extract from untreated samples procyanidins and catechin dominate.

Hierarchical clustering of the compounds classified as phenolic compounds, shown as heatmap in Supplementary Figure 3B, results in three main clusters. A clear separation between extracts from untreated and oven-treated samples with little differences according to the extraction solvent used in the first two clusters. The first cluster contains among others riboflavin, dihydroxyferulic acid, p-coumaric acid and cinnamic acid, compounds that have a higher MS-signal intensity in extracts from untreated BSG. The lower relative abundance of p-coumaric and cinnamic acid in dried samples is in line with the known thermo-instability of hydroxycinnamic acids (108), a downtrend also observed in temperature exposed broccoli, spinach and kale (109). The second cluster contains among others caffeic acid, hydroxybenzoic acid and hydroxybenzaldehyde, compounds that have higher signals in extracts from oven-treated samples. The increased abundance of these compounds was also found following the exposure of BSG to rising temperatures (110). This suggests that the drying process influences their release from the plant matrix or that they are formed through thermal degradation of more complex phenolic compounds. In the third cluster, variations between extracts from wet and dried BSG are notable, but the different solvents also had a considerable impact on the clustering. Compounds as apigenin, scoparin, tricin and oligomers thereof, (epi)gallocatechin, catechin, procyanidin dimer and trimer and saponarin gave a higher signal in 40%EtOH extracts compared to water and 20%EtOH extracts for both oven-treated and untreated BSG. The signal intensity of tricin and its derivatives were not affected by the heat treatment, while catechins and oligomers thereof (procyanidin dimer and trimer and (epi)gallocatechin-(epi)catechin) showed a lower signal in extracts from dried BSG. The instability of these latter compounds to different conditions of temperature and pH are known (111–113) and explains their lower signal intensity in dried BSG. The different impact of the pre-treatment and extraction solvent on compounds classified as phenolic compounds was highlighted by looking at which of the compounds contribute most to the accumulated signal for this group in extracts made with 40%EtOH (Figure 5). While in extracts from fresh BSG catechin and its oligomers represent 56% of the total signal intensity for the phenolic group, their relative contribution decreases in extracts from dried BSG. This decrease, attributed to the above-mentioned known thermolability of these compounds, makes that saponarin and tricin derivatives make up for most of the accumulated signal intensity in extracts from dried BSG (51%).

**Figure 6:**
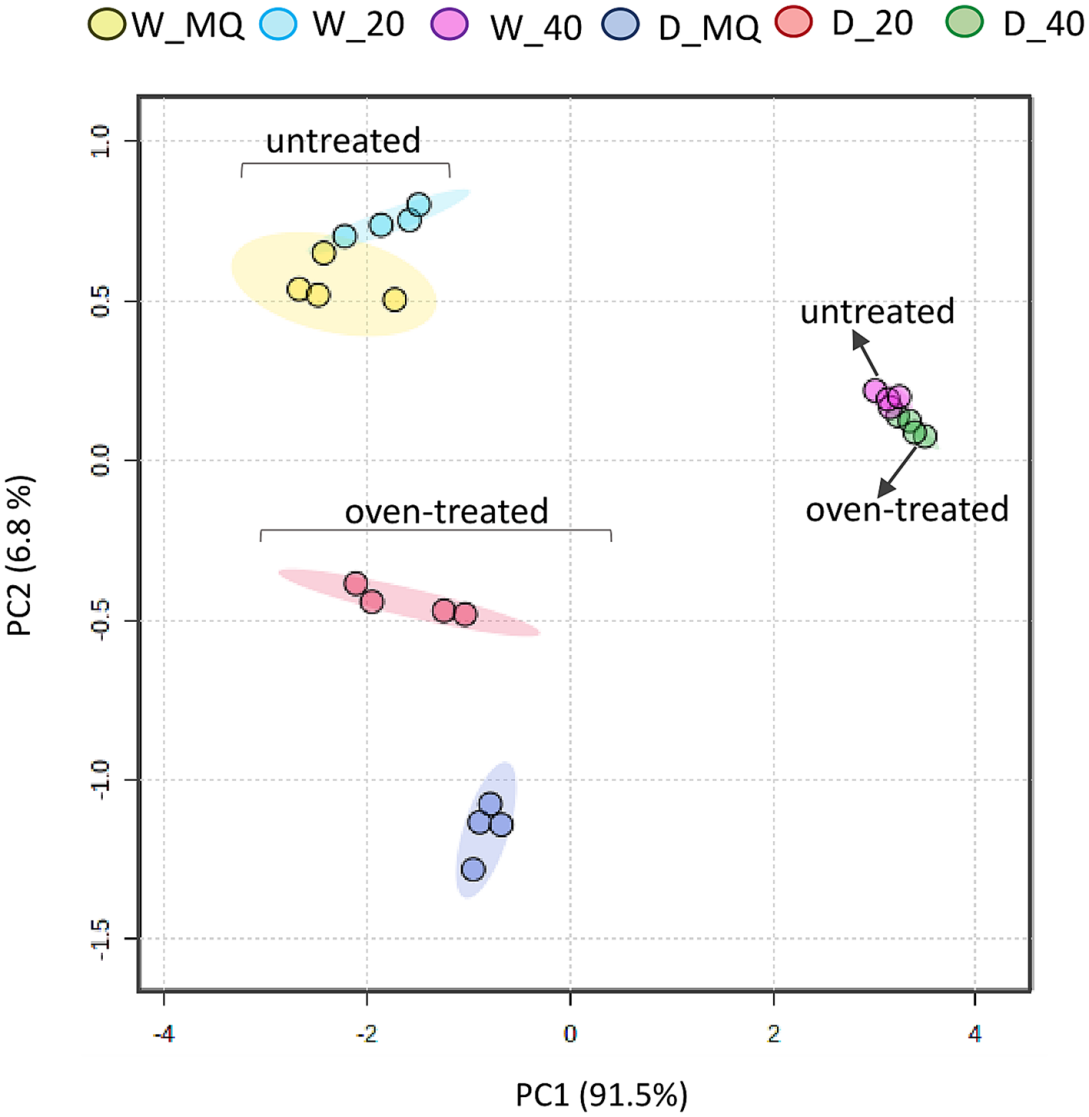
Principal component analysis (PCA) of lipids. W indicates extracts from wet, untreated BSG; D indicates extracts from ground oven-treated BSG. The annotations MQ, 20 and 40, respectively, indicate extracts obtained with MillQ water, 20% EtOH, and 40% EtOH. The PC1 covers more than 90% of the variabilities among all the samples. A complete separation is shown on the PC1 between the samples extracted with MillQ water and 20% EtOH from extracts with 40% EtOH. Samples are influenced to a lesser extent by the treatment of the starting material.

Lipids are the last group of compounds in this analysis, they account for 10% of the dried BSG biomass (15), and include phospholipids, fatty acids, mono- and di- and triglycerides (114). The principal lipids identified in this study were phospholipids, fatty acids, mono- and diglycerides and their galactosylated forms. Phosphatidylcholine (PC) and phosphatidylethanolamine (PE) with linoleic and palmitic acid were the most frequently identified lipids, as previously reported (115). Among the fatty acids present in BSG extracts (16, 116), linoleic acid was the most detected in this study. In addition, several hydroxylated fatty acids were also detected mainly based on C18 presenting one or two degrees of unsaturation (Hydroxyoctadec-n,n-enoic acids: HOME, DiHOME, TriHOME; HODE, DiHODE, TriHODE; HOTRE). As could be expected, extracts with 40%EtOH resulted in the highest MS intensity signals, and the extraction solvent is identified as the dominant determinant in the PCA with PC1 representing 91.5% of the total variance found in the dataset (Figure 6). Comparing untreated and oven-dried BSG at 40%EtOH, glycated lipids were detected in higher amounts in dried samples, while phospholipids were more present in extracts from untreated BSG. Hydroxy-fatty acids showed similar relative intensity signals between both types of starting materials (Supplementary Figure 3C). Owing to the high lipid content of BSG and the known disease-preventing and health promoting activities of some of the identified lipids, lipophilic extracts from BSG can be considered as good candidates for cosmetic, medical and nutraceutical applications (15).

These results demonstrate that the impact of oven-drying on the composition of BSG extracts is compound class–specific. The abundances of sugars, amino acids, polyamines, Amadori products, and peptides were predominantly affected by the drying treatment. The observed increases in Amadori products and short peptides are consistent with previous studies, reflecting heat-induced transformations coinciding with decreases in sugars and amino acids. These differential abundance changes make that targeted optimization of extraction parameters can be envisioned to either preserve or enrich extracts with specific compounds. For phenolic compounds, both the extraction solvent and drying treatment influenced their levels, with solvent being the main determinant and the heat effect varying by compound identity.

## 4 Conclusion

Our data provide preliminary evidence addressing the central scientific question of this study, drying of BSG prior to extraction may be revisited as our data show that it negatively affects several currently used quality parameters for BSG extracts. Under the tested laboratory conditions, extracts from fresh, unstabilized BSG showed higher biological antioxidant capacity and TPC, and a higher or similar abundance of compounds currently targeted in BSG valorisation studies, indicating that fresh BSG could be an advantageous feedstock for the food sector.

Further study is however needed. Firstly, the metabolomic data are based on relative MS signal intensities and level 2 compound identification, as is typical of an untargeted approach. Although such approach has the benefit of generating a global overview with a reasonable investment, confirmation based on the absolute quantification of key metabolites using analytical standards is needed to provide a more in-depth view on compositional differences between extracts from fresh and dried BSG. Furthermore, only absolute quantification would allow to assess how BSG extracts relate to extracts from other biomasses. Secondly, this study was conducted on a laboratory scale on a single BSG batch and should therefore only be considered as proof-of-concept. Prior to any real-life use, upscaling needs to be optimized and the results need to be confirmed with different batches of BSG. Finally, the main quality criteria of the current study were antioxidant capacity and TPC, also used as primary targets in most of the studies on BSG valorisation cited above. However, as our data show the range of compounds that are extracted is broad and higher relative abundances of compounds or compound classes are found in extracts from dried BSG. Therefore, depending on the envisioned application other bio-activity assays need to be implemented to find the best combination of pretreatment, extraction and down-stream processing.

Despite these limitations, together with the predictable and non-seasonal availability of BSG, the here presented data represent an encouraging starting point for future investigation into the exploitation of non-stabilized biomass at larger scale. Key aspects including microbial stability, storage performance, biochemical activity and scale-up feasibility remain to be evaluated to fully assess the potential of this approach.

## Data Availability

The original contributions presented in the study are included in the article/Supplementary material, further inquiries can be directed to the corresponding author.

## Footnotes

1
https://www.fao.org/3/cb4477en/online/cb4477en.html#figure_20_tab

2
https://hmdb.ca/spectra/ms/search

3
www.lipidmaps.org/

4
https://gnps.ucsd.edu/ProteoSAFe/libraries.jsp

5
www.mzcloud.org

6
https://pubchem.ncbi.nlm.nih.gov

7
https://metlin.scripps.edu/index.php

8
https://ipb-halle.github.io/MetFrag/

9
https://cfmid.wishartlab.com/

10
https://www.metaboanalyst.ca/MetaboAnalyst
