# Supplementary material for: Influence of oven-drying pre-treatment on the metabolic profile of extracts from brewer’s spent grains
Source: Front Nutr. 2026 May 29;13:1833417. doi: 10.3389/fnut.2026.1833417 (PMC13260077; doi:10.3389/fnut.2026.1833417)
Supplement: Supplementary file 1 [file Data_sheet_1.pdf]

Supplemental figure 1: Antioxidant capacity (FRAP) and total phenolic content (TPC) on BSG extracts

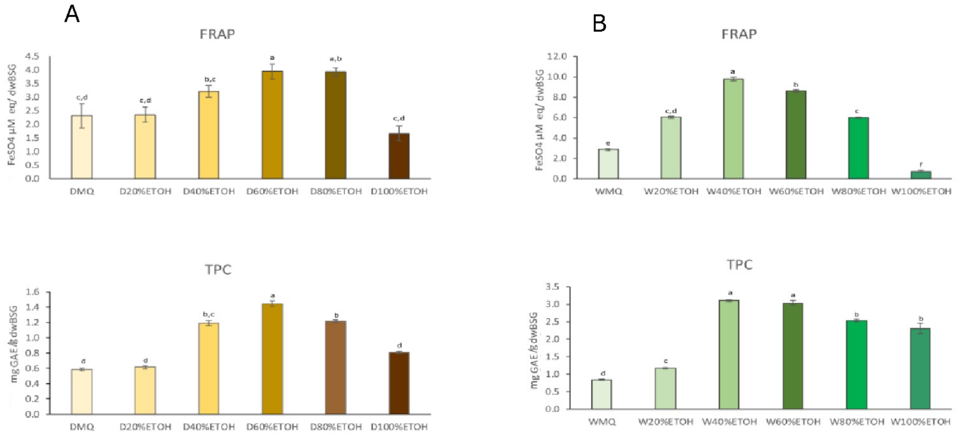

Total phenolic content (TPC) (Folin-Ciocalteu); antioxidant capacity (FRAP). D indicates extracts from ground oven-treated BSG (yellow, A) and W indicates extracts from wet, untreated BSG (green, B). The annotations MQ, 20, 40, 60 and 100 respectively indicate extracts obtained with MillQ water, 20% EtOH, 40% EtOH, 60% EtOH and 100% EtOH in a 1/20 BSG/solvent ratio.

Supplemental figure 2: Glucose assay on BSG extracts

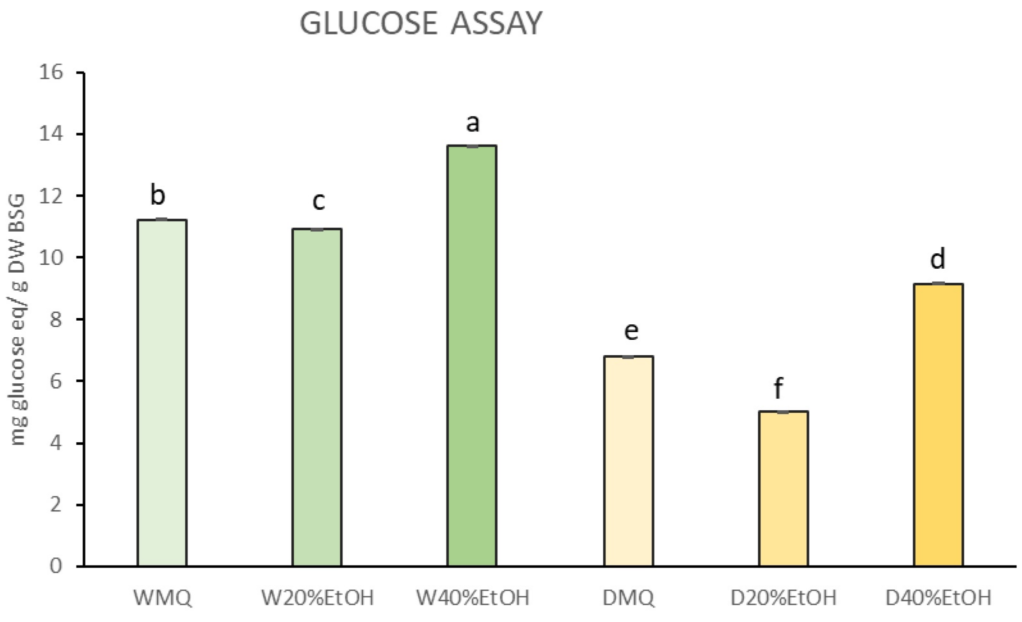

Glucose measurement on BSG extracts. D indicates extracts from ground oven-treated BSG (yellow) and W indicates extracts from wet, untreated BSG (green). The annotations MQ, 20 and 40respectively indicate extracts obtained with MillQ water, 20% EtOH and 40% EtOH in a 1/20 BSG/solvent ratio.

Supplemental figure 3A: Heatmap model of the identified amino acids in BSG extracts

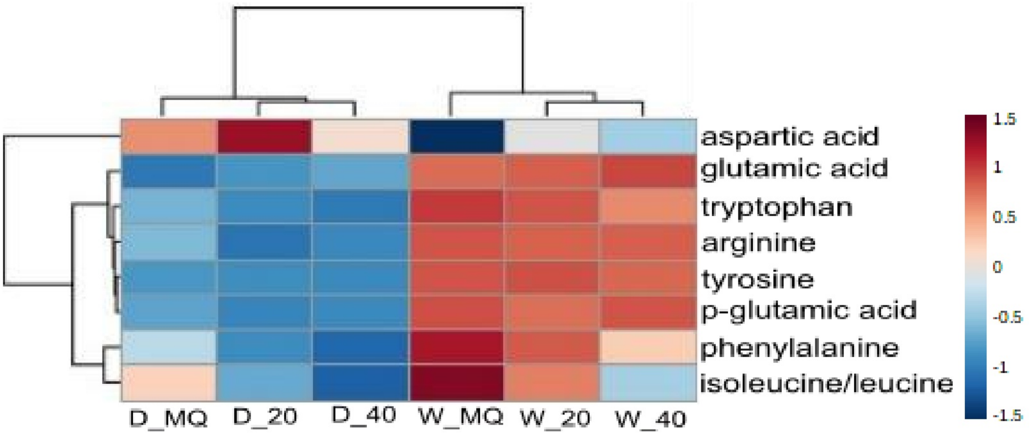

Heatmap model of the identified amino acids and their relative abundance. W indicates extracts from wet, untreated BSG; D indicates extracts from ground oven-treated BSG. The annotations MQ, 20 and 40 respectively indicate extracts obtained with MillQ water, 20% EtOH and 40% EtOH.

Supplemental figure 3B: Heatmap model of the identified phenolic compounds in BSG extracts

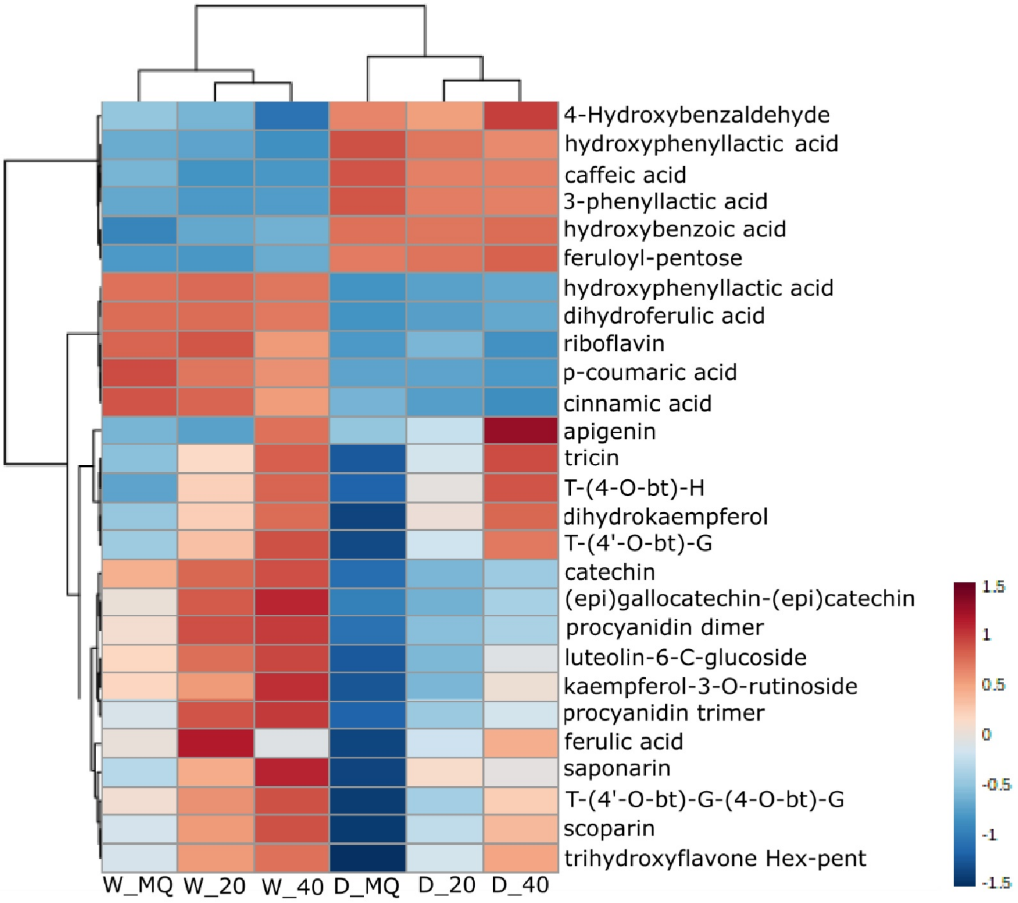

Heatmap model of the identified phenolic compounds and their relative abundance. W indicates extracts from wet, untreated BSG; D indicates extracts from ground oven-treated BSG. The annotations MQ, 20 and 40 respectively indicate extracts obtained with MillQ water, 20% EtOH and 40% EtOH

Supplemental figure 3C: Heatmap model of the identified groups of lipids in BSG extracts

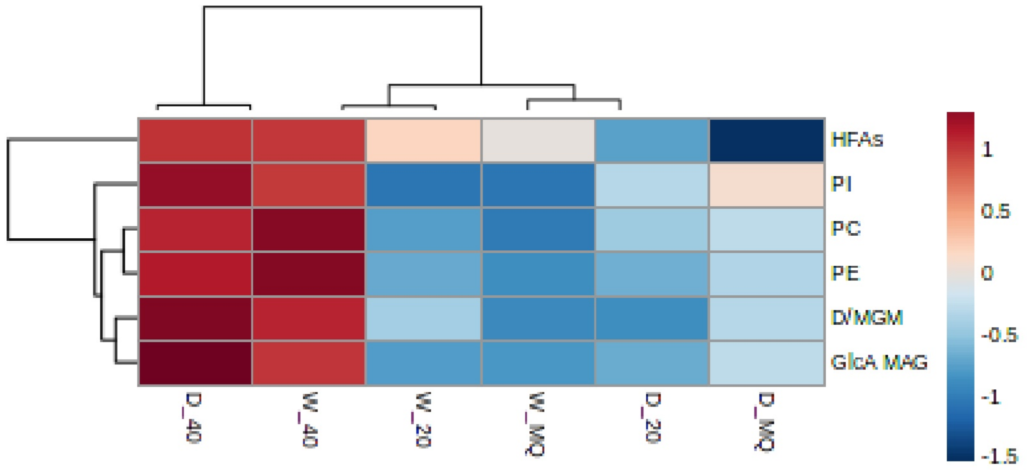

Heatmap model of the identified lipids and their relative abundance. W indicates extracts from wet, untreated BSG; D indicates extracts from ground oven-treated BSG. The annotations MQ, 20 and 40 respectively indicate extracts obtained with MillQ water, 20% EtOH and 40% EtOH. The annotations HFAs, PI, PC, PE, D/MGM and GlcA MAG respectively indicate hydroxy-fatty acids, phosphatidylinositol, phosphatidylcholine, phosphatidylethanolamine, di/monogalactosylmonoacylglycerol and glucuronosylmonoacylglycerols
